# Smell in Health and Disease

**Published:** 1906-08-04

**Authors:** 


The Hospital

A Journal of

The Medical Sciences and Hospital Administration.

Vol. XL.—No. 1,037. SATURDAY, AUGUST 4, 1906.

### Smell in Health and Disease.

Many startling stories are told about the sense of smell, partly because little is known about it, and partly because it is intimately connected with the cerebral hemispheres. It is clear that each individual has a distinctive odour of his own, apart from the eulogies of poets like Herrick, and of satirists like Martial. The traditional sweet smell of Alexander the Great is well known by Plutarch's account of that hero " whose skin," he says, " had a marvellous good savour, and his breath was very sweet, insomuch that his body had so sweet a smell of itself that all the apparel he wore next unto his body took thereof a passing delightful savour, as if it had been perfumed." In like manner, though it was more in the nature of a miracle, St. Francis de Paul, after he had subjected himself to frequent discipline and a 38 days' fast, exhaled a most sensible and delicious odour. Bromidrosis, on the other hand, is a well-known condition, and is consistent with health. Henry of Navarre was so great a sufferer that it was painful to his courtiers to come near him; and Mary of Medicis, his wife, used to smother herself and her rooms with floral essences when he approached. Negroes and the lower race of Chinamen have distinctive odours, and the meat-eating European is said to smell very offensively to the natives of countries in which a vegetarian diet predominates.

The odours attaching to disease are numerous, and in some cases distinctive. The smell of small-pox is not easily forgotten: the evil aroma of the surgical wards in a hospital can still be recalled very vividly by those who worked in them, even as late as 1880. An occasional case of gangrene brings back the old scenes of the pre-Listerian days with wonderful force. A persistent cadaveric smell, which may, however, be an hallucination, is sometimes a premonitory symptom of toxic absorption after a post-mortem examination. Gout, icterus, and cholera are said to have their peculiar odours, and the mouse-like smell of some forms of acute septic poisoning is well recognised. The sweat in dysentery has often the same smell as the dejecta, and malarial patients during their attacks sometimes think that they can smell the place where they were originally infected. The smell of freshly-plucked feathers is with less certainty said to attach to some cases of measles; that of bread hot from the oven to scarlatina; and the smell of mould to eczema and impetigo. The smell of acetone is now well recognised in the breath of patients suffering from diabetes who are on the verge of coma; and a similar apple-like and ethereal smell is not uncommon in children who are suffering from dyspepsia due to pancreatic insufficiency. Many observers have called attention to the distinctive smell of patients suffering from the plague, a smell which has been described by some as sour, and by others as apple-like. The smell of enteric, too, is known to those who have had to nurse during a typhoid epidemic, and it seems to be especially attractive to flies, which will congregate round a typhoid patient in countless numbers, though before his advent they were not specially numerous. The curious psychical effects of stimulating the sense of smell are also well known. It is still a moot point as to whether snuff-takers do not really stimulate their intellectual centres through the anterior lobes of their cerebral hemispheres when they irritate the olfactory mucous membrane. A habit so widely spread and so extensively practised as that of snuff-taking can hardly be supposed to depend entirely upon mimicry. There seems to be no doubt that the presence of some animals—the cat is the best-known instance—is really disturbing to persons of unusually acute sensibility, even when they are ignorant of its presence. In like manner many people regard as unpleasant, or even insupportable, odours like those of apples or pinks which are regarded as grateful by the majority of their fellows. Hammond, quoted by Gould in his " Anomalies and Curiosities of Medicine," tells an extraordinary story in connection with the effect of disorders of the nervous system associated with peculiar odours. A young woman of hysterical tendencies exhaled the odour of violets. The odour was given off from the left half of the chest only, and could be obtained concentrated by collecting the perspiration on a handkerchief, heating it with four ounces of spirit, and distilling the remaining mixture. The administration of salicylate of soda modified in some degree this violaceous odour.

